# Assessing the costs of GHG emissions of multi-product agricultural systems in Vietnam

**DOI:** 10.1038/s41598-022-20273-w

**Published:** 2022-10-28

**Authors:** Aito Yamamoto, Thi Kim Uyen Huynh, Yoko Saito, Takashi Fritz Matsuishi

**Affiliations:** 1 Graduate School of Global Food Resources, Hokkaido University, Kita9 Nishi9, Kita-ku, Sapporo, Hokkaido 060-8589 Japan; 2 Department of Economics in College of Economics, Can Tho University, 73/16/34, Nguyen Trai Street, Ninh Kieu District, Can Tho City, Vietnam; 3 Research Faculty of Agriculture, Hokkaido University, Kita9 Nishi9, Kita-ku, Sapporo, Hokkaido 060-8589 Japan; 4 GCF, Faculty of Fisheries Sciences, Hokkaido University, 3-1-1, Minato-cho, Hakodate, Hokkaido 041-8611 Japan

**Keywords:** Climate sciences, Environmental social sciences

## Abstract

Besides a vital sector of the economy, agriculture is a primary source of greenhouse gas (GHG) emissions. The present paper investigates the impact of carbon tax policy on Vietnamese agriculture by focusing on multi-product systems such as rice, livestock, and aquaculture, traditionally called the Vuon (Garden)–Ao (Pond)–Chuong (livestock pen) system (VAC). In it, farmers use garden, pond, and pen by-products as fertilizer and feed. We use shadow prices and Morishima substitution elasticities as greenhouse gas emissions indicators, estimated with directional output distance function. Farmers in the Mekong Delta region are found to be technologically less efficient than in other regions of Vietnam, though the shadow prices of GHG emissions are lower there too. This indicates that farmers in the Mekong Delta, generally concentrating either on livestock or aquaculture, have greater potential for reducing GHG emissions by way of improvements in technical efficiency than do those in other regions. However, Morishima elasticity estimates show that policy impacts diminish more quickly in the Mekong than elswhere. We suggest the Vietnamese government encourage Mekong Delta farmers to employ technologically more efficient methods or shift to more balanced farming to reduce the shadow price of GHG emissions, encouraging more efficient emissions reduction.

## Introduction

Reductions in greenhouse gas (GHG) emission from the agricultural sector, especially in developing countries, is an especially important approach to controlling global warming. The worldwide agricultural sector accounts for 20% of total anthropogenic emissions, of which 42% is methane (CH_4_) and 75% nitrous oxide (N_2_O)^1^. These agricultural gases are emitted largely from developing countries, who contribute to 80% of global agricultural emissions (https://www.fao.org/faostat/en/#data/GT)^2^. A government report of Vietnam (https://unfccc.int/resource/docs/natc/vnmbur1.pdf)^3^ indicates GHG emissions from agriculture comprise 33.2% of the national total. Vietnamese agriculture remains a primary GHG emission source because agricultural production was increasing at a 9.3% annual rate between 1991 and 2018 (https://www.fao.org/faostat/en/#data/QV)^4^.

Asian economies depend highly on agriculture. For instance, agriculture’s share of GDP is 14.9% in Vietnam, much higher than in the European Union, where it is only 1.7%^5^. Southeast Asian agricultural systems differ significantly from those in developed Western nations. In 2018 the United States earned only 0.6% of its agricultural output value (excluding aquaculture) from rice, but most of it from livestock and crops, while rice accounted for 22.3% (https://www.fao.org/faostat/en/#data/QV)^4^ of agricultural output value in Vietnam. In developed countries agricultural production is for commercial use, while in Vietnam and other Asian countries rice production is essential for household consumption too.

Vietnamese farmers have traditionally produced a large variety of products, using for instance the Vuon (Garden)–Ao (Pond)–Chuong (livestock pen) system. In the VAC system farmers can produce crops, livestock, and aquaculture not only for direct consumption or sale, but in their by-product uses as fertilizer and feed. For instance, the integrated pig-aquaculture system reduces fertilizer use in aquaculture^6^ and the integrated cattle-crop cultivation system improves productivity in both, boosting farmer income^7^. This multi-product system can thus be crucial for local food security and sustainable production^8^. Vietnam’s farm technology however will likely change drastically with economic development. Owing to relatively stable rice prices and less labor-intensive production technologies^9^, rice production has grown. But because land and labor productivity in livestock and aquaculture production are higher than they are in rice^10^, both livestock and aquacultural output production are expected to rise in the near future.

Unfortunately, Vietnamese agriculture is particularly vulnerable to climate change^11^, ranked 6th among the most seriously affected countries^12^, and GHG emissions may worsen this climate change impact. Severe droughts occurred during the 1997–2016 El Niño^13^, especially in the Central Highlands, and there was considerable salt-water intrusion in coastal provinces of the South Central and Mekong Delta regions. Vietnam’s Disaster Management Authority ()^14^ reports at least 60 storms or typhoons produced significant injury to the Vietnam economy from 2006 through 2019 (Table 1) and could bring losses in Vietnam’s total gross domestic product (GDP) through 2050^15^.

**Table 1:** Annual loss due to natural disasters in Vietnam.

	GDP (billion VND)	Total loss (million VND)	Share of total loss in GDP (%)
2006	1,061,565	18,565,661	1.75
2007	1,246,769	11,520,197	0.92
2008	1,616,047	13,299,389	0.82
2009	1,809,149	23,667,053	1.31
2010	2,739,843	16,062,290	0.59
2011	3,539,881	13,506,774	0.38
2012	4,073,762	15,935,421	0.39
2013	4,473,656	27,852,561	0.62
2014	4,937,032	2,828,348	0.06
2015	5,191,324	8,113,995	0.16
2016	5,639,401	39,726,339	0.70
2017	6,293,905	59,959,892	0.95
2018	7,009,042	20,000,000	0.29
2019	7,707,200	6,862,775	0.09
2020	8,044,386		

Annual Report^14^.

Severe droughts have led to salinity intrusion^16^ and farmers resort to aquaculture to counter this damage. Annual household income generated by the shrimp-rice rotation system is approximately 50% higher than in a double-cropping rice system. In shrimp-rice rotation, shrimp production is alternated with rice, while the double-cropped rice system can produce several types of paddy twice a year. Vietnam aquaculture accounted for 5.04% of total world production in 2018 and is the most important exporter of these products^17^.

This does not imply aquaculture has been environmentally friendly in Vietnam. On account of continuous flooding and double or triple cropping^18^, methane gas emissions from rice production are already higher there than in other countries such as China. Continued conversion of rice paddies into aquacultural ponds will worsen these emissions^19^. Environmentally optimal agricultural practices such as alternating wet and dry rice production (AWD)^20^ or a rice intensification system (SRI)^21^ will be the key to Vietnam’s future economic opportunities.

In recent years “carbon pricing” systems, which internalize the price of carbon, have been highlighted as an effective means of achieving environmental goals. The carbon tax should be one of the most effective policy options, as it offers emitters the flexibility to choose the most efficient method to meet their reduction targets^22^. This policy scheme alters farmer behavior by way of such economic incentives as tax reduction linked to environmentally efficient technology adoption^23^.

Theoretically, equalizing the marginal abatement cost among various economic entities is the most cost-minimizing option for reducing pollution^24^. Thus, from the policy perspective, estimating the marginal abatement cost (shadow price) of an undesirable output is crucial for implementating policy. However, a marginal abatement cost is often heterogeneous across firms^25,26^, implying contributions to emission reduction differ also. Specifically, lower-cost firms contribute more to emission reduction by assuming a larger reduction burden than the others do^27^. Understanding the marginal abatement costs of individual firms therefore is important from a policy perspective and is an empirical question.

Marginal costs of abatement of GHG emissions have been empircally estimated in several industrial sectors such as iron and steel industries, the most energy-intensive one being in China^28^ and the water supply industry in the UK^29^. It also is estimated at a provincial level in China, and the increasingly costly industrial SO_2_ emission reduction there has been detected^30^.

In the agricultural sector, marginal abatement costs have been estimated and compared by agricultural practice and land use. Comparison among such tillage practices as conventional and conservational forms has highlighted the opportunity cost of carbon sequestration^31^. Bio-economic modeling is used to calculate the opportunity costs of on-farm emissions, though results are expressed as *average* abatement costs^32^. Given the food security objectives in Norway, GHG reduction there can be achieved by altering peatland uses^33^. The shadow prices of three GHG gases in sheep-meat production has also been identified via a by-product model^34^. In Australia, farm-level marginal abatement cost has been estimated for multi-crop broadacre agriculture^25^ and dryland farming systems^35^. Some have discussed agricultural GHG mitigation potential from both an economic and environmental perspective^36,37^.

To the best of our knowledge however, none have yet considered the cost of environmental impacts of multi-crop agricultural systems in developing countries. And given their environmental as well as social and economic advantages, it is useful to evaluate the multiproduct rice, livestock, and aquaculture systems in particular. Though agricultural systems in Southeast Asian countries such as Thailand or Cambodia are similar to one another, we choose Vietnam as a model region because of the massive climate change impact on agriculture there. Vietnam has always ranked higher in the Climate Risk Index^12^ than Thailand does, and social and economic damages may be greater there given the future its agricultural sector most likely faces.

Our study focuses on impacts of the carbon tax policy on Vietnam agriculture with an emphasis on its multi-product agricultural systems, and is the first attempt to identify an economically and environmentally efficient Vietnam GHG emissions reduction policy at the farm level.

## Model and results

### Estimation strategy

#### Farmer incentive alignment with greenhouse gas reduction policy

**Figure 1:**
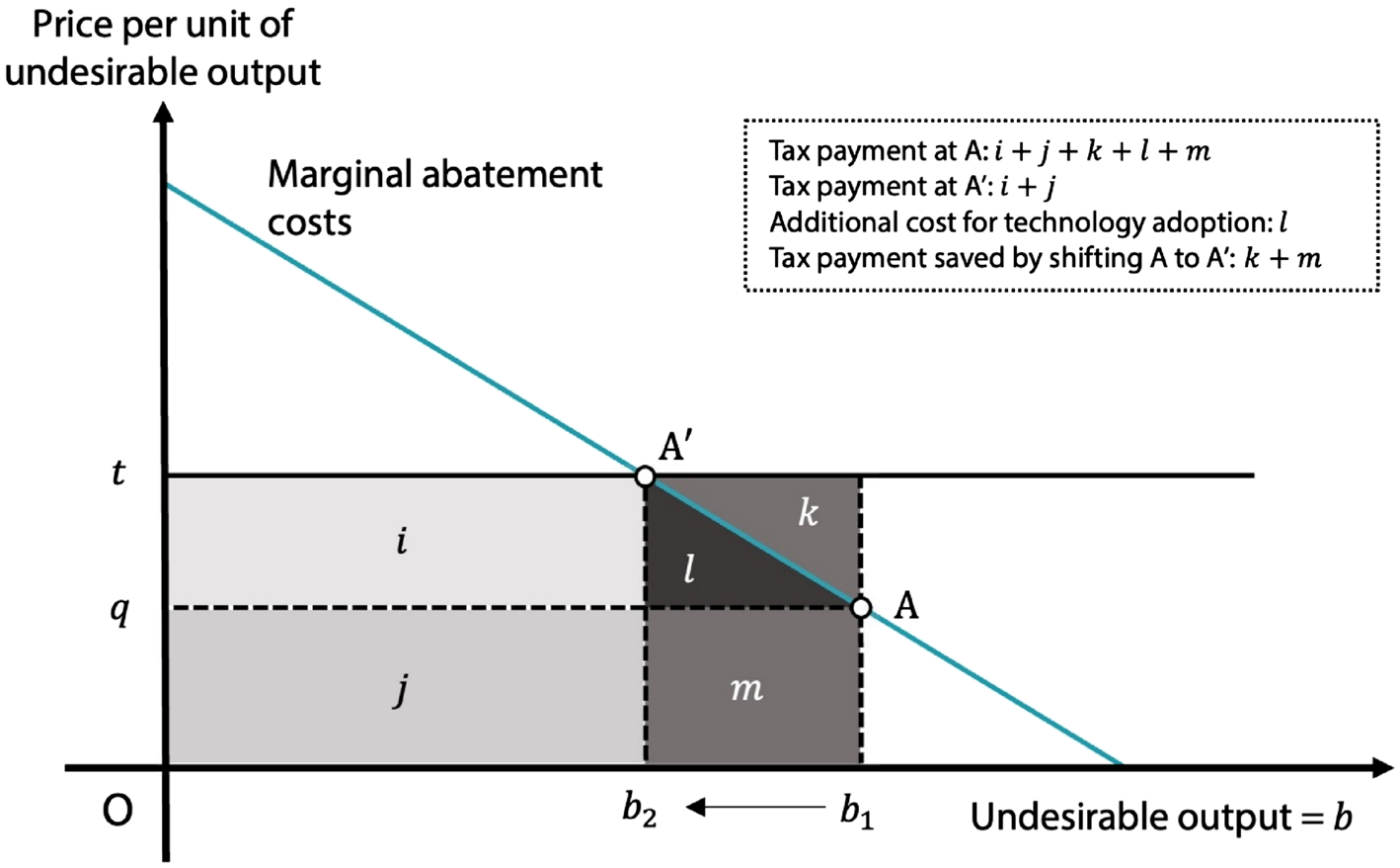
Structure of emission abatement costs.

In this section we describe farmer behavioral changes when such policies as environmental taxes are adopted to reduce agricultural pollutant emissions. In Fig. 1 we assume a farmer performs at point A where the shadow price of the undesirable output *q* is emitted in quantity ${b}_{1}$. The shadow price is the opportunity cost, in terms of a contracted desirable output, of reducing a pollutant by an additional unit^38^. It is used frequently by policymakers to formulate environmental taxes and emissions trading indicators^39,40^.

Suppose a carbon tax policy is introduced at $t$ per unit of undesirable output (Fig. 1). At this point, the farmer’s tax payment is ${b}_{1}*t (=i+j+k+l+m)$. Under this scenario, the tax rate exceeds the farmer’s own shadow price (*t* > *q*); thus, the policy provides an incentive to shift the production pattern to an environmentally efficient point A’ given the farmer’s choices are profit-maximizing.

By moving from A to A’ in the figure, the tax rate is equated to the farmer’s shadow price (*t* = *q*), resulting in cost of $l$ reducing the undesirable output from *b*_*1*_ to ${b}_{2}$ because farmers use environmentally more efficient production technologies such as the AWD or SRI systems mentioned above. Technology adoption is costly but farmers can save on tax payments in the amount *k* + *m* because taxes are levied only on a GHG emissions up to ${b}_{2}$. The total tax farmers must pay is ${b}_{2}*t (=i+j)$ in Fig. 1. As long as the cost of technology adoption is less than the tax farmers can save, that is *l* < (*k* + *m*), farmer’s net benefit of reducing GHG emissions is positive. Thus, the carbon tax provides an incentive to reduce GHG emissions up to the point where the shadow price of undesirable output coincides with the tax rate.

#### Shadow pricing model of undesirable outputs

The present study uses the distance function approach to estimate a pollutant’s shadow price^41^. Traditionally the Shepherd distance function has been used to measure a business entity’s efficiency or productivity^42^. This approach varies both desirable and undesirable outputs in proportion to their initial size, often inappropriate from a policy standpoint. Directional distance functions have thus been developed to remain consistent with the mix of the policy maker’s present situation and intentions, so that desirable outputs are expanded and undesirable outputs contracted in the directions and proportions the policy maker wishes to explore. This method has been used frequently in environmental sensitivity analysis^43,44^. The directional output distance function allows calculation of an undesirable output’s shadow price on the basis of its duality with the entity’s revenue function^45^, convenient because revenue data tend to be more accessible than cost data. We therefore adopt the directional output distance function approach to calculate GHGs’ shadow price and investigate farmer emission reduction behaviors.

We first consider a production process of rice, livestock, and aquaculture as a vector of desirable outputs $y=({y}_{1}, \dots , {y}_{M})\in {R}_{+}^{M}$, and greenhouse gas emissions as undesirable outputs $b=({b}_{1}, \dots , {b}_{J})\in {R}_{+}^{J}$ in the presence of a vector of inputs $x=({x}_{1}, \dots , {x}_{N})\in {R}_{+}^{N}$ such as capital, land, and labor.

The agricultural production technology $P\left(x\right)$ is then defined as the convex output set satisfying:

1 $$\begin{array}{c}P\left(x\right)=\left\{\left(y,b\right):x\;can\;produce\;\left(y,b\right)\right\}.\end{array}$$

The boundary of $P\left(x\right)$ is the Pareto efficient frontier^45^. Thus the general directional output distance function is defined as^46^

2 $$\begin{array}{c}{\overrightarrow{D}}_{o}\left(x,y,b; {g}_{y},-{g}_{b}\right)=max\left\{\beta :\left(y+\beta {g}_{y}, b-\beta {g}_{b}\right)\in P\left(x\right)\right\},\end{array}$$

where $\left({g}_{y},-{g}_{b}\right)$ is a vector setting the direction of output changes and so providing the maximum expansion of the desirable outputs and contraction of the undesirable ones. The directional output distance function reflects each sample farm’s inefficiency. The lower its value the more efficient the farm, and the higher the value the more inefficient.

**Figure 2:**
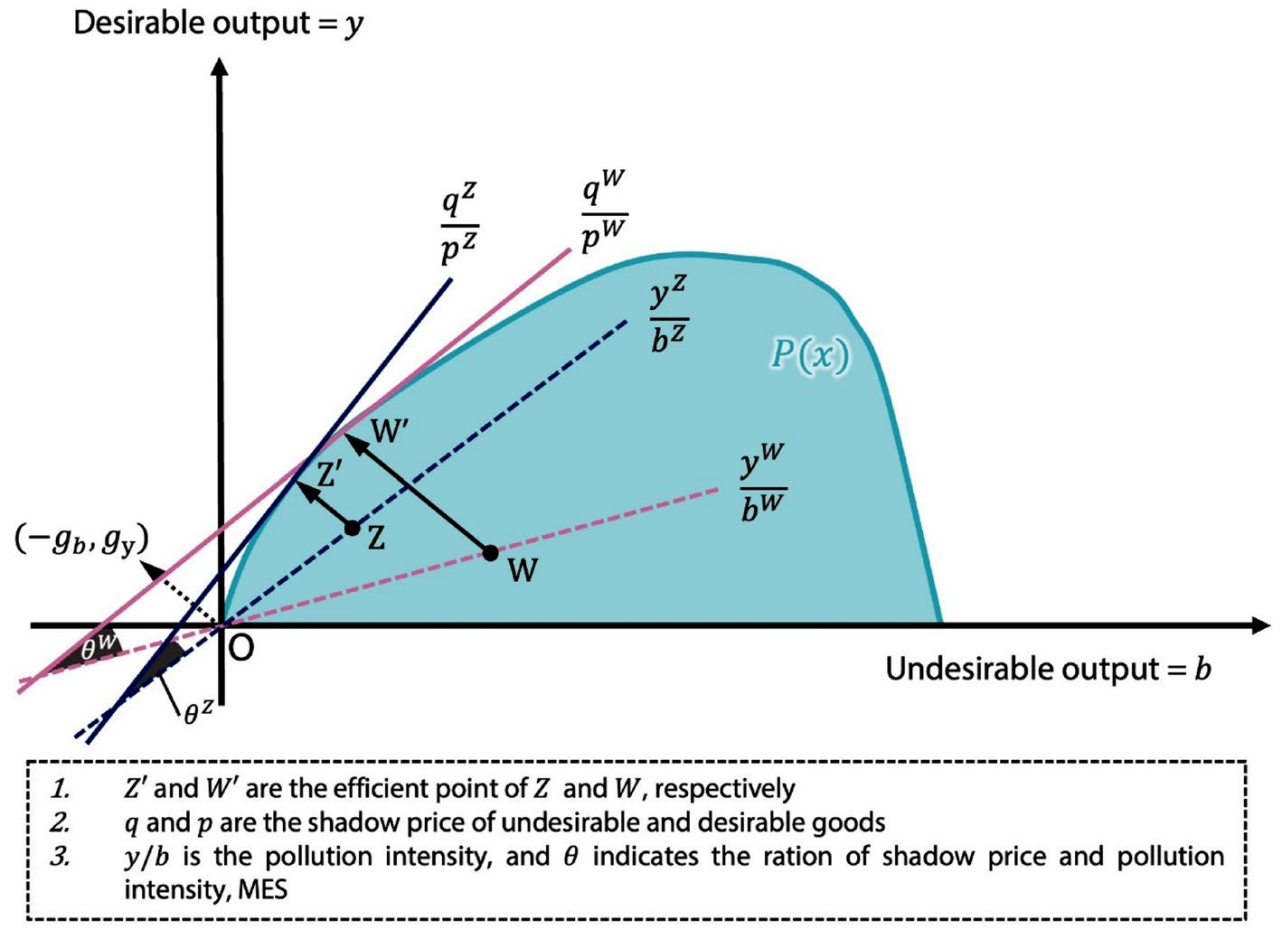
Output set (directional output distance) and associated shadow pricing.

Let us assume two farmers are producing at points Z and W respectively (see Fig. 2). The distance from the production frontier is longer for the farmer at W, implying point W is less efficient than point Z is, which is situated at a shorter distance to the efficiency frontier. Lines tangent to the frontier are expressed as $q/p$, where $p$ is the market price per unit of desirable output and $q$ the shadow price per unit of undesirable output. In particular shadow price $q$ is computed as the cost of reducing one unit of *undesirable* output at the loss of the *desirable* output. Figure 2 demonstrates a case in which W’s shadow price (at W′) is lower than that of Z (at Z′).

The ratios $y/b$ and $q/p$ represent the Morishima elasticities of substitution ($MES$) when given in the elasticity form. The MES can be used to measure how the undesirable/desirable shadow price ratio ($q/p$) changes as relative pollution intensity (ratio $y/b$ of desirable to undesirable output) changes. A negative MES indicates raising pollution intensity $y/b$ becomes more costly as the shadow price ratio ($q/p$) rises. A positive value implies raising the desirble output is associated with the decrease of undesirble outputs^29,30^.

If the shadow price equals $t$ in Fig. 1, there is no incentive to reduce the undesirable output. At point ($q<t$) in contrast, there is an incentive to reduce it to save on tax payments. In other words, we can improve an inefficient agricultural system by setting a tax rate higher than the shadow price. Boosting the MES’s absolute value, however, exhausts a policy intervention’s advantages at an early stage because the shadow price’s proportional increase here is high. Therefore, each farmer’s shadow price and substitution elasticity estimate provide important information on environmental policy applications. Through them we can investigate the farmer’s behavior in shifting from inefficient to more efficient methods and the corresponding possibilities of greenhouse gas reduction.

### Regional differences

**Figure 3:**
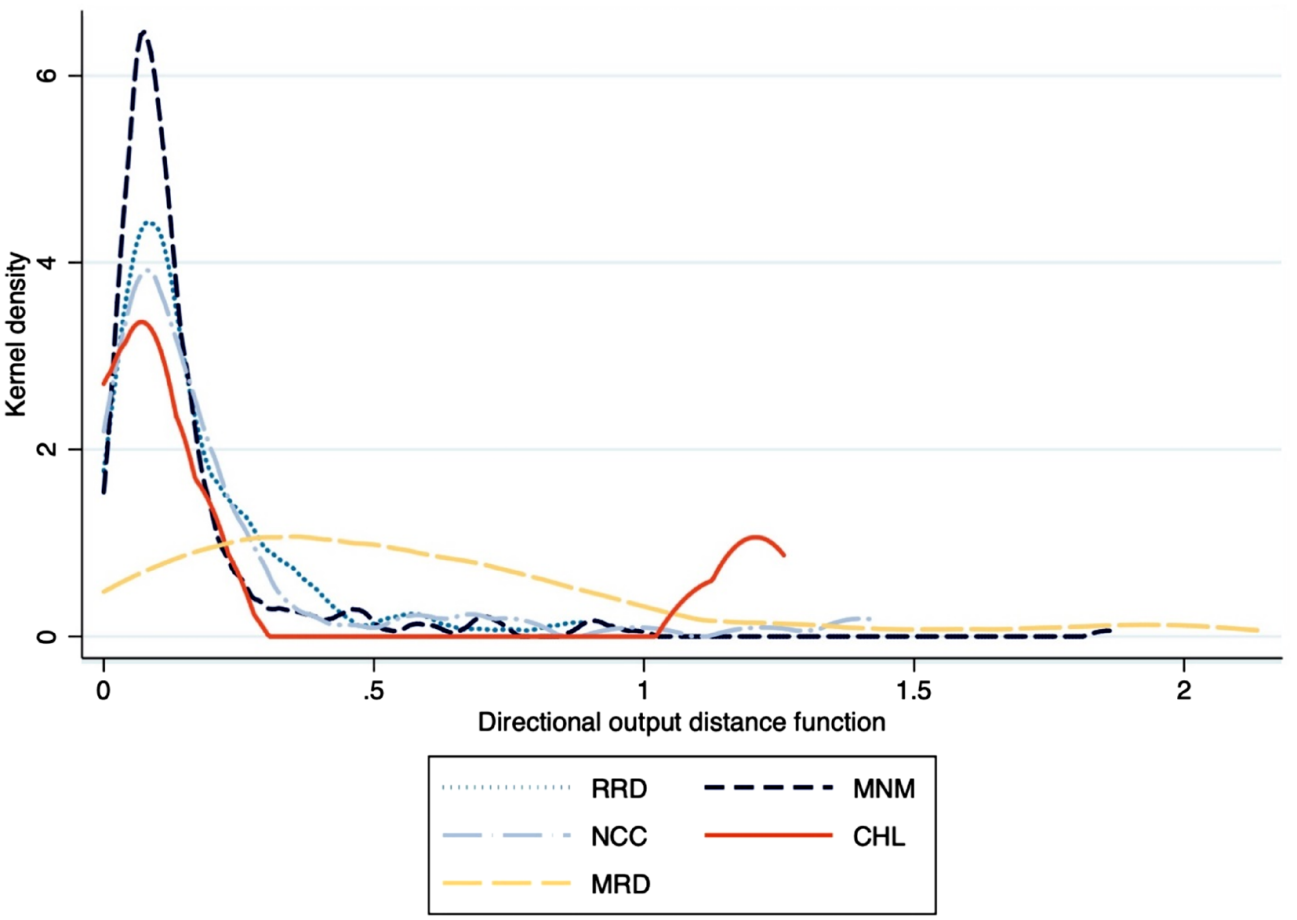
Kernel probability densities of directional output distance, by region.

Figure 3 provides a plot of the region-specific kernel probability densities of directional output distance, that is of technical inefficiency. The density in the southeast has been removed because contained only one household. The Red River Delta in the north, midlands and northern mountains (MNM), the northern and central coast (NCC), and central highlands (CHL) show relatively greater chances of technical efficiency, that is of operating a shorter distance to the technical frontier, than the Mekong Delta (MRD) does, where high technical inefficiencies are widespread. Wilcoxon rank-sum tests confirm this on the present densities (MRD vs. CHL, p = 0.017; MRD vs. the other three, p = 0.000). Mekong farmers are significantly less technically efficient than in any other region, implying great potential for reducing greenhouse gas emissions through technical efficiency improvements.

In terms of desirable vs. undesirable outputs (Fig. 2), Mekong River farmers are operating at production point W and the northern regions at point Z. The high dykes under construction in the Mekong area since the early 2000s have produced soil degradation, so that more fertilizer and pesticide are required to support the three-intra-year rice cultivation system employed there (in particular, respectively 54–87% and 62% higher than in the system with lower dykes)^47^. In areas with the older, lower dykes (usually more than 15 years old), fertilizer input rose by 133–234% and pesticide input by 118%^47^. More inputs are being needed on account of the soil loss, implying inefficient production and high pollution costs.

**Figure 4:**
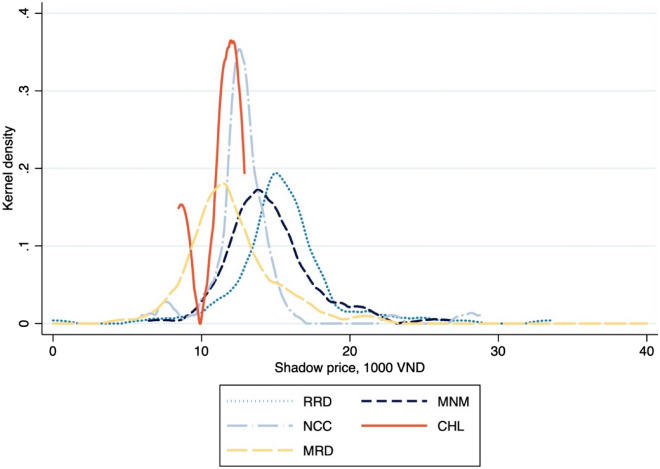
Kernel probability densities of shadow-price in rice production, by region.

Rice’s shadow-price kernel densities are shown in Fig. 4. No significant difference is found in the density structures between the Mekong and the Central Highlands (p = 0.615), whereas differences are present between the Mekong and the northern and coastal area (p = 0.004), the Red River Delta (p = 0.000), and the midlands and northern mountains (p = 0.000). Shadow prices themselves are significantly higher in the north than in the Mekong, implying the cost of reducing GHG releases in the north will be higher also. In other words, northern farmers must sacrifice more desirable goods, something less true for farmers in the southern areas once the carbon tax is implemented.

**Figure 5:**
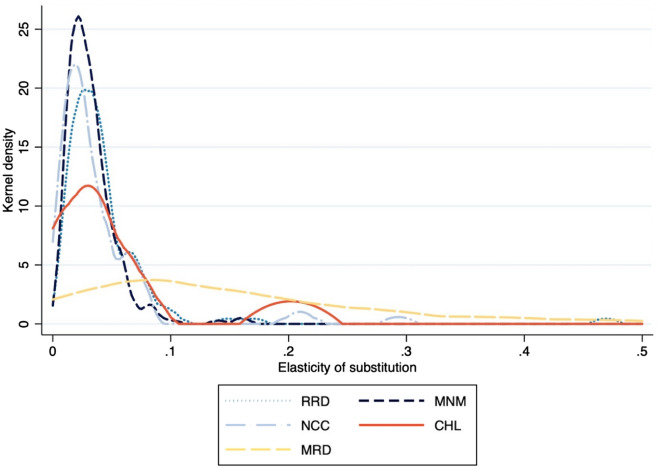
Kernel probability densities of Morishima substitution elasticity in rice production, by region.

Table 2 provides our Morishima substitution elasticities, and Fig. 5 the corresponding probability density curves for rice. An MES can be negative or positive, a negative value indicating emission reduction is costly^48,49^ and a positive value that more desirables will be possible by reducing the undesirables^29,30^. We find a positive MES in rice cultivation and negative one in livestock and aquaculture. Though it is low everywhere, its positive value in rice production indicates that in Vietnam at least, reducing GHG emissions is consistent with producing more rice. In every Vietnam region, the negative MES in livestock operations is in absolute value greater than it is in aquaculture. This coincides with findings that livestock is normally the larger greenhouse gas emitter^50,51^, so that all else equal, aquaculture might be preferred over livestock.

**Table 2:** Morishima elasticity by region.

	RRD (n = 104)		MNM (N = 254)		NCC (n = 72)	
	Mean	Std. dev	Mean	Std. dev	Mean	Std. dev
M_GHG, rice_	0.044	0.050	0.032	0.022	0.038	0.047
M_GHG, livestock_	− 0.076	0.125	− 0.060	0.074	− 0.063	0.089
M_GHG, aquaculture_	− 0.030	0.074	− 0.013	0.041	− 0.014	0.028

	CHL (n = 9)		MRD (n = 75)			
	Mean	Std. dev	Mean	Std. dev		
M_GHG, rice_	0.049	0.061	0.162	0.160		
M_GHG, livestock_	− 0.057	0.087	− 0.087	0.121		
M_GHG, aquaculture_	− 0.019	0.020	− 0.043	0.102

On the other hand, the MES’s kernel probability density in Mekong rice production (Fig. 5) is distributed well to the right of the other regions’, implying a higher mean substitution between desirables and undesirables there than elsewhere in the country. The Wilcoxon rank-sum test shows this difference (MRD and CHL, p = 0.002; MRD and the three other regions, p = 0.000) is statistically significant. That is, increasing pollution intensity (*y/b*) is possible there—by reducing the shadow price of the undesirable output—at *less* cost than it is elsewhere.

### Differences in production systems

We now categorize farm households by the share of livestock revenue in combined livestock and aquaculture production (i.e. excluding rice), because livestock and aquaculture are both expected to expand on account of their high productivity and rising export demand. We will take balanced farming to mean that a balance is taking place between livestock and aquaculture as well as between that pair and rice production—while unbalanced means production leans toward either livestock or aquaculture.

Table 3 shows three separate indicators of balanced *vs* unbalanced farming: the directional output distance function, the shadow price, and the MES. In the Wilcoxon rank-sum test result in the last column of the table, the output distance is significantly lower in balanced farming (0.092) than it is in unbalanced (0.136), implying balanced farming is the technically more efficient of the two. Balanced-farming’s shadow price (14.47) is also significantly higher than unbalanced-farming’s (13.34), reasonably implying that the opportunity cost of reducing GHG emissions is the more costly when farming is balanced, that a greater loss of desirable output occurs than when farming is unbalanced. Reducing GHG emissions will naturally become less costly at the margin once unbalanced farming becomes converted to a more balanced way of improving technical efficiency. No significant change (p-value = 0.082) however is expected in the MES.

**Table 3:** Descriptive statistics, by production system, between livestock and aquaculture.

	Unbalanced farming		Balanced farming		
	n = 330		n = 205		
	Median	Interquartile range	Median	Interquartile range	p-value
Directional output distance function $({\overrightarrow{D}}_{o})$	0.136	0.225	0.092	0.099	0.000
Shadow price $({q}_{GHG})$	13.337	3.615	14.474	2.926	0.000
Morishima elasticity of substitution $({MES}_{b,y})$	0.034	0.035	0.029	0.029	0.082

Unbalanced farming indicates that livestock revenue accounts for 0–25% or 75–100% of the partial revenue of livestock and aquaculture, excluding rice. Balanced farming indicates that livestock or aquaculture accounts for 25–75%.

The synergistic effect of combined cropping and livestock improves resource use efficiency^8^. In aquaculture ponds, yield per unit area is highest because of the nutrient circulation from growing a variety of products^52^. The implication is that farmers utilizing balanced methods have the higher resource use efficiency and productivity because nutrient circulation is greater.

In general, aquaculture production might be recommendable over livestock^50,51^. But it is not necessarily more effective to lift aquaculture production when an operation is unbalanced than when it is balanced. This is because the two types of unbalanced farming—the livestock intensive (75–100%) and the aquaculture intensive (0–25%)—show no significant difference per indicator (p > 0.05), shown in Table 1A (Supplementary Table 1A).

## Discussion

A regional assessment of our results indicates that the southern region, especially the Mekong Delta, has achieved the lowest technical efficiency and shadow price in the country so can reduce greenhouse emissions at the least cost—because in doing so it improves farm technical efficiency. In Fig. 1, point A could represent the Mekong, earning a lower price on its undesirable outputs than the other regions do at A′.

Our analysis of the alternative production systems shows farmers following a balanced livestock-aquaculture production enjoy the greatest technical efficiency and shadow prices. The carbon tax policy provides incentives for farms with unbalanced systems to reduce GHG emissions by shifting to a balanced one.

Our elasticity-of-substitution (MES) estimates in the Mekong reveal high responsiveness in all three combinations. Rather elastic negative MES values in livestock and aquaculture show the rate of increase in shadow price of GHG emissions to be high, while positive values in rice production suggest a decrease. Since 68% of farms in the Mekong are operating in more unbalanced manner than the other regions, shifting to balanced farming by boosting rice production is the less costly way to reduce emissions. On the other hand, high output productivity in and strong foreign demand for livestock and aquaculture discourage balancing and raise the shadow price of GHG emissions sharply.

Given the negative and rather elastic MES in livestock and aquaculture in the Mekong, shadow prices there are such that efficient point $t$ in Fig. 1 will be achieved with an undesirable-output reduction lower than it will in the other provinces. Emission reduction in the Mekong, that is, ceases at a point higher than *b*_*2*_, implying an intervention policy’s effectiveness in the Mekong will be exhausted earlier than elsewhere. If emission control requires further reduction to point *b*_*2*_, the Mekong region has two options. Since the tax becomes cheaper than the shadow price, buying an emission permit will be attractive if a trading system for doing so is available. Another possibility is to adopt technology or such agricultural practices as balanced farming in a multi-product system.

Although the Vietnamese government has decided to implement an emissions trading scheme (ETS; https://www.eastasiaforum.org/2020/11/19/vietnam-pioneers-post-pandemic-carbon-pricing/#more-313603)^53^, management problems have surfaced^54^. A carbon tax is an efficient way to reduce greenhouse gases but carbon pricing interventions have significant negative implications for per-capital GDP, stifling Vietnam’s economic growth^55^. Since emission reduction costs may be higher in some farm households than in others, government should think about using these tax revenues to compensate the farmers involved. Equating the returns of a per-capita carbon tax across households will contribute to environmental goals and improve well-being, help eliminate inequality, and alleviate poverty^56^.

Aquaculture plays an important role in food security and export markets in Vietnam as well as elsewhere in Southeast Asia, so is important to consider in an emissions reduction plan. This study has assessed the advantages of multi-crop production and of including aquaculture in it. Yet aquaculture is rarely considered in the GHG mitigation policy process.

Vietnam is north–south elongated, influencing climate and optimal productions systems. Environmental policies need to take this agricultural diversity into account and resist policies modeled directly after developed nations, where systems are large, uniform, and commercial.

Owing to data limitations, GHG emissions have been estimated here on the basis of a production system’s emission intensity (EI). More refined emissions data will be useful in the formulation of still more specific policies.

## Methods

### Data

For household data we use the 2016 Vietnam Household Living Standard Survey (VHLSS) provided by the General Statistics Office of Vietnam. The VHLSS covers 46,995 households in 3,133 communes/wards. The present study focuses on 537 farming households that produce rice, livestock, and aquaculture, given the regional differences in their production patterns. Pollution reduction cost varies by region and production system, suggesting targets that vary by region^57^. This we account for, focusing on the RRD, MNM, NCC, CHL, SEA, and MRD (Supplementary Fig. 1A). Results provide insights into the likely effects of introducing a carbon tax to Vietnamese agriculture, which vary by both production system and region, to achieve a more sustainable agriculture.

We use the general algebraic modeling system (GAMS) with the CPLEX solver to estimate the directional output distance function. To avoid convergence problems, all input and output variables are normalized by their mean values^43,49^. We thus focus on a hypothetical farm household that uses the sample mean quantities of inputs and to produce the sample mean quantities of outputs, (*x*, *y*, *b*) = (1, 1, 1). Supplementary Table 4A shows the parameter estimates used to identify the directional output distance function. In the process of parameter estimation, we dropped the 0.37% (2/537) of the observations that violated constraints (ii) and (iv) below.

The data used in this study are randomly and independently selected. We employ the Wilcoxon rank-sum test, which satisfies the following conditions: (1) the shape of the probability distribution is unknown; (2) it is compared between two groups with no correspondence; and (3) the test is robust against outliers.

### Directional output distance function

To estimate the shadow prices of the undesirable outputs, we take advantage of the duality between the directional output distance function and the revenue function, given all input and output prices^39,58^. The revenue function is defined as^45^:

3 $$\begin{array}{c}R\left(x, p, q\right)={max}_{y,b}\left\{py-qb:\left(y, b\right)\in P\left(x\right)\right\},\end{array}$$

where $x=({x}_{1}, \dots , {x}_{N})\in {R}_{+}^{N}$ is the set of inputs, $p=({p}_{1}, \dots , {p}_{M})\in {R}_{+}^{M}$ the set of outputs, and $q=({q}_{1}, \dots , {q}_{J})\in {R}_{+}^{J}$ their quantities. ${R}_{+}^{N},$ ${R}_{+}^{M}$ and ${R}_{+}^{J}$ are sets of positive real numbers. $P\left(x\right)$ is the set of outputs producing desirable (y) and undesirable (b) outputs. Under the assumption of g-disposability of $\left(y-{g}_{y}, b+{g}_{b}\right)\in P\left(x\right)$, where ${g}_{y}, {g}_{b}$ are the directional vectors of desirable and undesirable outputs respectively, the revenue function can be rewritten as

4 $$\begin{array}{c}R\left(x, p, q\right)={max}_{y,b}\left\{py-qb: {\overrightarrow{D}}_{o}\left(x,y,b; g\right)\ge 0\right\} ,\end{array}$$

where ${\overrightarrow{D}}_{o}\left(x,y,b; g\right)$ is the directional output distance function. We assume weak disposability in desirable and undesirable outputs because reducing GHG emissions involves such costs as reducing inputs that are the sources of emissions but whose use had earned a net return. Given directional vector $\left({g}_{y},-{g}_{b}\right)$ that allows for simultaneous expansion of desirable outputs and contraction of undesirable ones, the revenue function is depicted in the form:

5 $$\begin{array}{c}R\left(x, p, q\right)\ge \left(p, -q\right)\left[y+{\overrightarrow{D}}_{o}\left(x,y,b; g\right) {\cdot g}_{y}, b-{\overrightarrow{D}}_{o}\left(x,y,b; g\right) {\cdot g}_{b}\right] \\ \iff R\left(x, p, q\right)\ge \left(py-qb\right)+p{\overrightarrow{D}}_{o}\left(x,y,b; g\right) {\cdot g}_{y}+q{\overrightarrow{D}}_{o}\left(x,y,b; g\right) {\cdot g}_{b}\end{array}$$

Equation (5) implies that maximum revenue is obtained by correcting technical inefficiencies, both in increasing desirable outputs $\left[p{\overrightarrow{D}}_{o}\left(x,y,b; g\right) {\cdot g}_{y}\right]$ and reducing undesirable ones $\left[q{\overrightarrow{D}}_{o}\left(x,y,b; g\right) {\cdot g}_{b}\right]$. Rearranging (Eq. 5), the directional output distance function can now be represented in terms of the revenue function as:

6 $$\begin{array}{c}{\overrightarrow{D}}_{o}\left(x,y,b; g\right)\le \frac{R\left(x, p, q\right)-\left(py-qb\right)}{p{g}_{y}+q{g}_{b}} \\ ={min}_{p, q}\left\{\frac{R\left(x, p, q\right)-\left(py-qb\right)}{p{g}_{y}+q{g}_{b}}\right\}\end{array}$$

### Shadow price

Twice applying the envelope theorem to Eq. (6), we obtain the following two first-order conditions for revenue maximization:

7a $$\begin{array}{c}{\nabla }_{b}{\overrightarrow{D}}_{o}\left(x,y,b; g\right)=\frac{q}{p{g}_{y}+q{g}_{b}} \ge 0,\end{array}$$

7b $$\begin{array}{c}{\nabla }_{y}{\overrightarrow{D}}_{o}\left(x,y,b; g\right)=\frac{-p}{p{g}_{y}+q{g}_{b}} \le 0.\end{array}$$

Using Eqs. (7a) and (7b), where the price of the $m$ th desirable output is ${p}_{m}$, the shadow price of greenhouse gas emission ${q}_{1}$ of the $j$ th undesirable output can be expressed as

8 $$\begin{array}{c}{q}_{1}=-{p}_{m}\left(\frac{\partial {\overrightarrow{D}}_{o}\left(x,y,b; g\right)/\partial {b}_{1}}{\partial {\overrightarrow{D}}_{o}\left(x,y,b; g\right)/\partial {y}_{m}}\right) , m=1, 2, 3.\end{array}$$

In this study the shadow price is calculated as farm-specific, that is ${q}_{1}=-{p}_{1}\left[\partial {\overrightarrow{D}}_{o}\left(x,y,b; g\right)/\partial {b}_{1}/\partial {\overrightarrow{D}}_{o}\left(x,y,b; g\right)/\partial {y}_{m}\right]$, where ${b}_{1}$ is the undesirable output or GHG emission and *y*_*m*_ the desirable ones, in our case rice, aquaculture, and livestock.

### Morishima substitution elasticity

When the shadow price is representable by Eq. (8), the Morishima elasticity of substitution between desirable and undesirable outputs is defined as^49^:

9 $$\begin{array}{*{20}c} {M_{{b,y}} = \frac{{\partial \ln (q_{1} /p_{m} )}}{{\partial \ln (y_{m} /b_{1} )}} = y_{m} ^{*} \left[ {\frac{{\partial ^{2} \overrightarrow {{D_{O} }} \left( {x,y,b;g} \right)/\partial b_{1} \partial y_{m} }}{{\partial \overrightarrow {{D_{O} }} \left( {x,y,b;g} \right)/\partial b_{1} }} - \frac{{\partial ^{2} \overrightarrow {{D_{O} }} \left( {x,y,b;g} \right)/\partial y_{m} \partial y_{m} }}{{\partial \overrightarrow {{D_{O} }} \left( {x,y,b;g} \right)/\partial y_{m} }}} \right],} \\ \end{array}$$

where ${{y}_{m}}^{*}={y}_{m}+\overrightarrow{{D}_{O}}\left(x,y,b;g\right)$. Morishima substitution elasticity ${M}_{b,y}$ indicates how the shadow price of GHG emissions would change if the farmer’s relative pollution intensity changed by one percent. If ${M}_{b,y}$ in (9) is negative, a trade-off is present between the desirable and undesirable outputs. If by contrast it is positive, desirable outputs can be expanded and undesirable outputs contracted simultaneously.

### Empirical model

To parameterize our directional output distance function we choose the quadratic functional form because, unlike the translog, it can be restricted to satisfy the translation property^45^. We set the directional vector to $g=\left(1,-1\right)$ for parsimonious parameterization. Under this condition it is also consistent with policymaker preference for expanding desirable outputs and contracting the undesirable ones. For these purposes we express the quadratic directional distance function as^45^

10 $$\begin{aligned} \vec{D}_{o} \left( {x_{k} ,y_{k} ,b_{k} ;1, - 1} \right) & = \alpha + \sum\limits_{{n = 1}}^{N} {\alpha _{n} } x_{{nk}} + \sum\limits_{{m = 1}}^{M} {\beta _{m} } y_{{mk}} + \sum\limits_{{j = 1}}^{J} {\gamma _{j} } b_{{jk}} \\ & \quad + \frac{1}{2}\sum\limits_{{n = 1}}^{N} {\sum\limits_{{n^{\prime} = 1}}^{N} {\alpha _{{nn^{\prime}}} x_{{nk}} x_{{n^{\prime}k}} } } + \frac{1}{2}\sum\limits_{{m = 1}}^{M} {\sum\limits_{{m^{\prime} = 1}}^{M} {\beta _{{mm^{\prime}}} y_{{mk}} y_{{m^{\prime}k}} } } + \frac{1}{2}\sum\limits_{{j = 1}}^{J} {\sum\limits_{{j^{\prime} = 1}}^{J} {\gamma _{{jj^{\prime}}} b_{{jk}} b_{{j^{\prime}k}} } } \\ & \quad + \sum\limits_{{n = 1}}^{N} {\sum\limits_{{m = 1}}^{M} {\delta _{{nm}} } } x_{{nk}} y_{{mk}} + \sum\limits_{{n = 1}}^{N} {\sum\limits_{{j = 1}}^{J} {\eta _{{nj}} } } x_{{nk}} b_{{jk}} + \sum\limits_{{m = 1}}^{M} {\sum\limits_{{j = 1}}^{J} {\mu _{m} } } y_{{mk}} b_{{jk}} , \\ \end{aligned}$$

where $k=1,\dots ,K$ are the households conducting the agricultural activity. Equation (10) then, can be expressed in terms of Eqs. (11a) and (11b) as:

11a $$\begin{array}{*{20}l} {\frac{{\partial \vec{D}_{o} \left( {x_{k} ,y_{k} ,b_{k} ;1, - 1} \right)}}{{\partial b_{1} }} = \gamma _{j} + \sum\limits_{{j^{\prime} = 1}}^{J} {\gamma _{{jj^{\prime } }} } b_{{j^{\prime } k}} + \sum\limits_{{n = 1}}^{N} {\eta _{{nj}} } x_{{nk}} + \sum\limits_{{m = 1}}^{M} {\mu _{{mj}} } y_{{mk}} \ge 0,} \hfill \\ \end{array}$$

11b $$\begin{array}{*{20}c} {\frac{{\partial \vec{D}_{o} \left( {x_{k} ,y_{k} ,b_{k} ;1, - 1} \right)}}{{\partial y_{m} }} = \beta _{m} + \sum\limits_{{m^{\prime} = 1}}^{M} {\beta _{{mm^{\prime}}} } y_{{m^{\prime}k}} + \sum\limits_{{n = 1}}^{N} {\delta _{{nm}} } x_{{nk}} + \sum\limits_{{j = 1}}^{J} {\mu _{{mj}} } b_{{jk}} \le 0,\quad m = 1,2,3.} \\ \end{array}$$

To control for province effects, we add provincial dummy variables to Eq. (10)’s intercept term as:

12 $$\begin{array}{c}\alpha ={\alpha }_{0}+\sum_{k=1}^{K-1}{\rho }_{{S}_{k}}{S}_{k},\end{array}$$

where ${\rho }_{{S}_{k}}$ is the coefficient of the regional variable ${S}_{k}$, namely ${S}_{k^{\prime}}=1$ if ${k}^{^{\prime}}=k$, and 0 otherwise.

To estimate parameters $\alpha _{0} ,\alpha _{n} ,\alpha _{{nn^{\prime}}} ,\beta _{m} ,\beta _{{mm^{\prime}}} ,\gamma _{j} ,\gamma _{{jj^{\prime}}} ,\delta _{{nm}} ,\eta _{{nj}} ,\mu _{{mj}} ,$ and ${\rho }_{{S}_{k}}$ in Eq. (10), we solve the linear program^30^:

13 $$\begin{array}{c}minimize \sum_{k=1}^{K}\left[{\overrightarrow{D}}_{o}\left({x}_{k},{y}_{k},{b}_{k}; 1, -1\right)-0\right]\end{array}$$

$$s.t.$$

(i) $${\overrightarrow{D}}_{o}\left({x}_{k},{y}_{k},{b}_{k}; 1, -1\right)\ge 0, k=1, \dots , K$$
(ii) $${\overrightarrow{D}}_{o}\left({x}_{k},{y}_{k},0; 1, -1\right)\le 0, k=1, \dots , K$$
(iii) $${\overrightarrow{\partial D}}_{o}\left({x}_{k},{y}_{k},{b}_{k}; 1, -1\right)/\partial {b}_{j}\ge 0, j=1, \dots , J;k=1, \dots , K$$
(iv) $${\overrightarrow{\partial D}}_{o}\left({x}_{k},{y}_{k},{b}_{k}; 1, -1\right)/\partial {y}_{m}\le 0, m=1, \dots , M; k=1, \dots , K$$
(v) $${\overrightarrow{\partial D}}_{o}\left(\overline{x },{y}_{k},{b}_{k}; 1, -1\right)/\partial {x}_{n}\ge 0, n=1, \dots , N; k=1, \dots , K$$
(vi) $$\sum\limits_{{m = 1}}^{M} {\beta _{m} } - \sum\limits_{{j = 1}}^{J} {\gamma _{j} } = - 1;\sum\limits_{{m^{\prime} = 1}}^{M} {\beta _{{mm^{\prime}}} } - \sum\limits_{{j = 1}}^{J} {\mu _{{mj}} } = 0,m = 1, \ldots ,M;$$
  $$\sum\limits_{{j^{\prime} = 1}}^{J} {jj^{\prime}} - \sum\limits_{{m = 1}}^{M} {\mu _{{mj}} } = 0,j = 1, \ldots ,J;\sum\limits_{{m = 1}}^{M} {\delta _{{nm}} } - \sum\limits_{{j = 1}}^{J} {\eta _{{nj}} } = 0,n = 1, \ldots ,N$$
(vii) $$\alpha _{{nn^{\prime}}} = \alpha _{{n^{\prime}n}} ,n \ne n^{\prime};\beta _{{mm^{\prime}}} = \beta _{{m^{\prime}m}} ,m \ne m^{\prime};\gamma _{{jj^{\prime}}} = \gamma _{{j^{\prime}j}} ,j \ne j^{\prime}.$$

Objective function (13) minimizes the sum of deviations in the distance between the efficient frontier and farm-household-level observations. Restriction (i) ensures that the production set is feasible. Restriction (ii), related to the null-jointness property, assures that $(y, 0)$ be non-feasible for any non-negative $y$. Monotonicity conditions are imposed by restrictions in (iii)–(v). Restrictions (vi) and (vii) respectively impose the translation property and symmetry conditions.

### Calculation of GHG emissions

The GHG emissions for farm $k$ are calculated from the EI as (https://www.fao.org/faostat/en/#data/EI)^59^:

14 $$\begin{array}{c}{GHG}_{k}= \sum_{i=1}^{I}{EI}_{i}\cdot {P}_{i,}\end{array}$$

where ${EI}_{i}$ and ${P}_{i}$ denote the EI in kg of CO_2_eq per kg of output $i$ and the quantity of production in kg of output $i$ respectively. Note that the production *quantity* is used in this GHG calculation while its *value* is used in the estimation of the directional output distance function. Supplementary Tables 2A and 3A give the definitions and descriptive statistics of all input and output data used in this study.

## Supplementary Information

Supplementary Information.

## Data Availability

The datasets supporting the findings of this study are available from the corresponding author upon request.
