# Supplementary material for: Assessing the costs of GHG emissions of multi-product agricultural systems in Vietnam
Source: Sci Rep. 2022 Oct 28;12:18172. doi: 10.1038/s41598-022-20273-w (PMC9616835; doi:10.1038/s41598-022-20273-w)
Supplement: Supplementary file 1 — Supplementary Information. [file 41598_2022_20273_MOESM1_ESM.docx]

# Supplementary Material

Table 1A | Descriptive statistics by production style, livestock and aquaculture intensive

|  | Livestock intensive | |  | Aquaculture intensive | | |  |  |
| --- | --- | --- | --- | --- | --- | --- | --- | --- |
|  | n=275 | |  | n=55 | | |  |  |
|  | Median | Interquartile range |  | | Median | Interquartile range |  | p-value |
| Directional output distance function $(\vec{D}_{o})$ | 0.133 | 0.228 |  | | 0.160 | 0.230 |  | 0.774 |
| Shadow price $(q_{GHG})$ | 13.397 | 3.623 |  | | 13.231 | 4.197 |  | 0.210 |
| Morishima elasticity  of substitution $({MES}_{b,y})$ | 0.033 | 0.033 |  | | 0.038 | 0.054 |  | 0.460 |
| Note:  Livestock intensive means that livestock revenue accounts for 75–100% of the partial revenue of livestock and aquaculture, excluding rice. Aquaculture intensive means that aquaculture accounts for 75–100% of the partial revenue. | | | | | | | | |

Table 2A | Definition of variables

| Variables | Description |
| --- | --- |
| Rice output | Total annual value of rice production |
| Rice price | Price per kg of rice per farmer, divided by the output value of the harvested quantity |
| Livestock output | Annual total value of livestock production: pork, beef, water, buffalo, goat, and sheep meat, and chicken |
| Aquaculture output | Annual total of the value of aquaculture production: fish and shrimp |
| Capital input | Total asset value: machine, equipment, and ploughing cattle |
| Land input | Cultivated area of rice |
| Labor input | Annual total labor days: family and hired labor |
| Materials & services (M&S) input | Annual total expenditure: fertilizer, fuel, repairs and maintenance, and feed |
| Greenhouse gas (GHG) output | Total annual on-farm emissions from rice, livestock, and aquaculture production. For rice and livestock, we use the emission intensity (EI) data from FAOSTAT for 2017^[59]^. For aquaculture, we use the EI of fish and shrimp in Southeast Asia for 2017, based on MacLeod et al.^[51]^. For fish, we used the mean value of the EI of four dominant species (i.e., Indian carp, Cyprinid, FW fish, and Catfish) farmed in Southeast Asia. Note that for livestock and aquaculture, the ratio of carcass weight is multiplied by the EI based on MacLeod et al.^[51]^ and Schweihofer^[60]^. |
| Note:  1) EI [kgCO_2_/kg product (CW)]: rice=0.9128, pork=1.6932, beef/buffalo=25.4941, chicken=1.2104, goat/sheep=33.3825, fish=1.60, and shrimp=7.48. Note that the value of buffalo is integrated into that of beef because the quantity of available beef is considered to exceed that of buffalos significantly.  2) CW ratio: pork=70%, beef/buffalo=62%, chicken=71%, goat/sheep=71%, fish=66%, and shrimp=50%. | |

Table 3A | Descriptive statistics

| Variable |  | Unit | Mean | Std. dev. | Min. | Max. |
| --- | --- | --- | --- | --- | --- | --- |
| *Inputs* |  |  |  |  |  |  |
|  | Capital $(x_{1})$ | 10^3^ VND | 4,911.4 | 10,966.8 | 40 | 185,061 |
|  | Land $(x_{2})$ | m^3^ | 7,887.8 | 14,320.1 | 360 | 195,255 |
|  | Labor $(x_{3})$ | Days | 628.9 | 354.3 | 40 | 2,834 |
|  | Materials and services $(x_{4})$ | 10^3^ VND | 36,003.8 | 81,037.7 | 945 | 947,810 |
| *Desirable outputs* | |  |  |  |  |  |
|  | Rice $(y_{1})$ | 10^3^ VND | 26,131.8 | 52,866.1 | 921 | 748,403 |
|  | Livestock $(y_{2})$ | 10^3^ VND | 32,932.9 | 102,975.2 | 270 | 1,788,035 |
|  | Aquaculture $(y_{3})$ | 10^3^ VND | 18,628.7 | 76,882.3 | 124 | 1,090,389 |
| *Undesirable outputs* | |  |  |  |  |  |
|  | Greenhouse gas emissions $(b_{1})$ | kg CO_2_eq | 5,500.6 | 10,209.4 | 361 | 146,703 |

Table 4A | Estimated parameters

| Parameter | Variable | Estimate |
| --- | --- | --- |
| $\alpha_{0}$ | Intercept | 0.0273 |
| $\alpha_{1}$ | $x_{1}$ | 0.0138 |
| $\alpha_{2}$ | $x_{2}$ | 0.0131 |
| $\alpha_{3}$ | $x_{3}$ | -0.0128 |
| $\alpha_{4}$ | $x_{4}$ | 0.0721 |
| $\beta_{1}$ | $y_{1}$ | -0.2751 |
| $\beta_{2}$ | $y_{2}$ | -0.0763 |
| $\beta_{3}$ | $y_{3}$ | -0.0537 |
| $\gamma_{1}$ | $b_{1}$ | 0.5949 |
| $\alpha_{11}$ | ${x_{1}}^{2}$ | 0.0115 |
| $\alpha_{12}$ | $x_{1}x_{2}$ | -0.0187 |
| $\alpha_{13}$ | $x_{1}x_{3}$ | 0.0104 |
| $\alpha_{14}$ | $x_{1}x_{4}$ | -0.0178 |
| $\alpha_{22}$ | ${x_{2}}^{2}$ | -0.0449 |
| $\alpha_{23}$ | $x_{2}x_{3}$ | 0.0089 |
| $\alpha_{24}$ | $x_{2}x_{4}$ | 0.0411 |
| $\alpha_{33}$ | ${x_{3}}^{2}$ | -0.0009 |
| $\alpha_{34}$ | $x_{3}x_{4}$ | -0.0056 |
| $\alpha_{44}$ | ${x_{4}}^{2}$ | -0.0404 |
| $\beta_{11}$ | ${y_{1}}^{2}$ | 0.0150 |
| $\beta_{12}$ | $y_{1}y_{2}$ | -0.0131 |
| $\beta_{13}$ | $y_{1}y_{3}$ | -0.0060 |
| $\beta_{22}$ | ${y_{2}}^{2}$ | -0.0038 |
| $\beta_{23}$ | $y_{2}y_{3}$ | -0.0058 |
| $\beta_{33}$ | ${y_{3}}^{2}$ | -0.0001 |
| $\gamma_{11}$ | ${b_{1}}^{2}$ | -0.0389 |
| $\delta_{11}$ | $x_{1}y_{1}$ | 0.0023 |
| $\delta_{12}$ | $x_{1}y_{2}$ | 0.0061 |
| $\delta_{13}$ | $x_{1}y_{3}$ | -0.0005 |
| $\delta_{21}$ | $x_{2}y_{1}$ | -0.0043 |
| $\delta_{22}$ | $x_{2}y_{2}$ | -0.0007 |
| $\delta_{23}$ | $x_{2}y_{3}$ | 0.0145 |
| $\delta_{31}$ | $x_{3}y_{1}$ | 0.0000 |
| $\delta_{32}$ | $x_{3}y_{2}$ | 0.0000 |
| $\delta_{33}$ | $x_{3}y_{3}$ | 0.0000 |
| $\delta_{41}$ | $x_{4}y_{1}$ | 0.0101 |
| $\delta_{42}$ | $x_{4}y_{2}$ | 0.0165 |
| $\delta_{43}$ | $x_{4}y_{3}$ | 0.0084 |
| $\eta_{11}$ | $x_{1}b_{1}$ | 0.0079 |
| $\eta_{21}$ | $x_{2}b_{1}$ | 0.0095 |
| $\eta_{31}$ | $x_{3}b_{1}$ | 0.0000 |
| $\eta_{41}$ | $x_{4}b_{1}$ | 0.0349 |
| $\mu_{11}$ | $y_{1}b_{1}$ | -0.0042 |
| $\mu_{21}$ | $y_{2}b_{1}$ | -0.0228 |
| $\mu_{31}$ | $y_{3}b_{1}$ | -0.0120 |


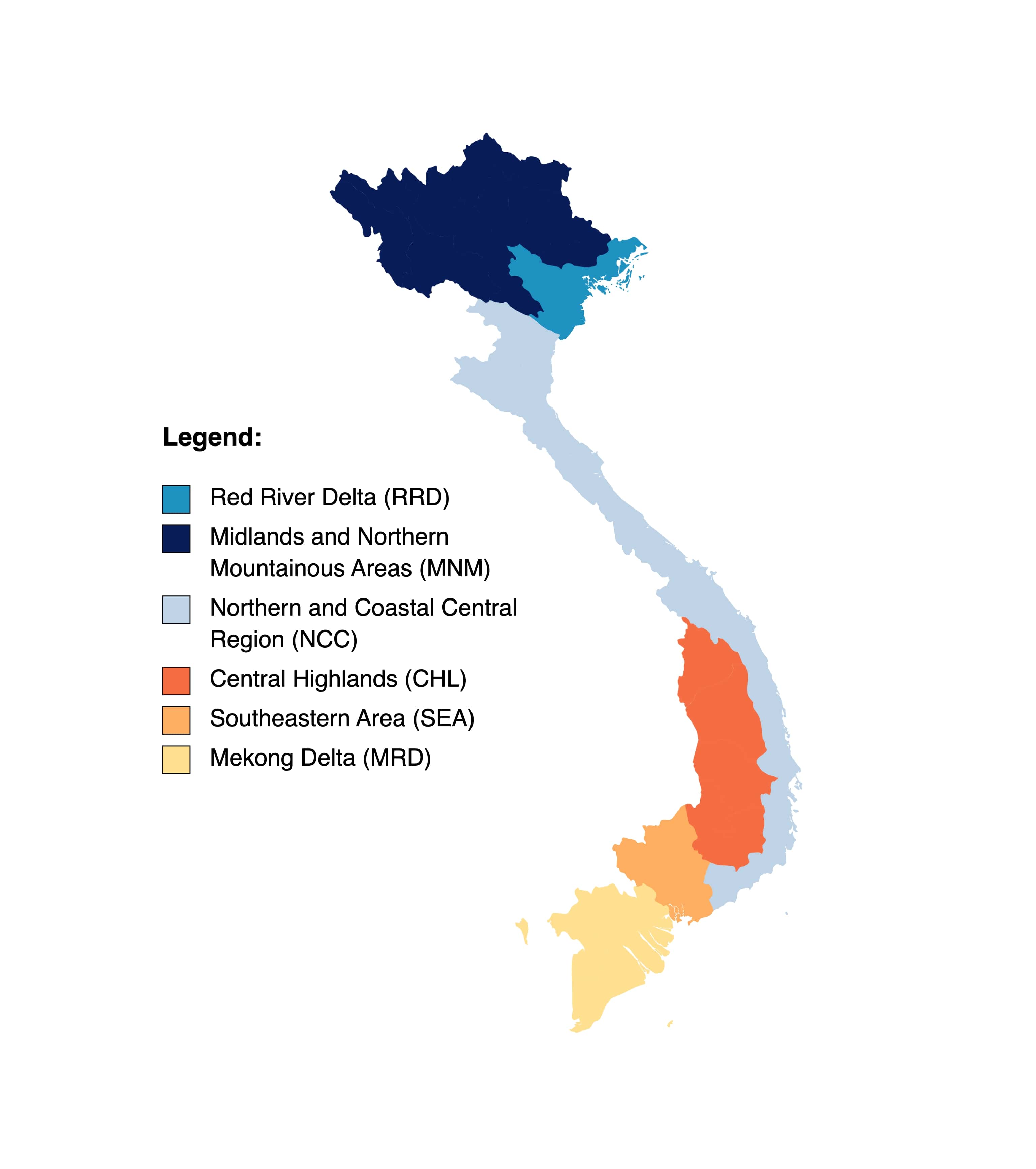


Fig. 1A | The six classified regions of Vietnam
